# Supplementary material for: Characterization of adenine phosphoribosyltransferase (APRT) activity in Trypanosoma brucei brucei: Only one of the two isoforms is kinetically active
Source: PLoS Negl Trop Dis. 2022 Feb 1;16(2):e0009926. doi: 10.1371/journal.pntd.0009926 (PMC8836349; doi:10.1371/journal.pntd.0009926)
Supplement: S1 Appendix — (PDF) [file pntd.0009926.s001.pdf]

S1 Appendix. Optimized sequences of APRT1 and APRT2, showing the ORFs used for heterologous expression in *P. pastoris*, with N and C terminal overhangs used for cloning into pPICZ vectors (underlined).

>APRT1_opt_Ntag

tgtacttccaaagcggtaccatgagtcttgttgaggttttgcctaactattttacattgtctaa
ggattcaccattgagaaagaagttcgagaaagtctataagtggtactctccagctttttctcct
catgatgttccaagattcgctgaagttggtaacattactgaaaatcctgaggttatgagaggta
tcagagatttctttgttgatagatacaagaacttgcaacaaccaatcactcacattttgggttt
tgattctagaggtttcttgttgggtcctatgattgctgttgaattgaacgttccattcgttttg
attagaaaggctaacaagattgctggtgttattattaagtctgagccttacactaaagaatatg
ctgctgagtctgaagagtgtatgactgttagattcggttctttcgataagaactctagagttgt
tttgatcgatgatgttattgctactggtggtactatgttggctggtgttcaattggttgatgct
tgtggtgctactttggttgaggttgctggtattttgggtttgactttcttgaaaggtactcaac
ctgctcatactttcgctggtggtagatactctaacgttccattcgttactttggttgatgaaac
tgttttgtctgatgagaattgtggtgacccattgcaccacaaaggaagtagaattattagttgc
gctgaagccaagaagttgatttgagtttgtagccttagaca

>APRT2_opt_Ntag

tgtacttccaaagcggtaccatgagtcagtatgatgctatccttaccgaaagacacccacacca
cttcacccttgccgacacccacccacttgctaaagaacttcacgctaacatttttggagagtct
gatttgactcatgctaattgtgctcacgtttacgatatttcttctttgactgaaaagccagctt
tgtttagaaaagttattgagttcttgaagtgtagatacgaaactatgggagatactggtcctac
tcatattattggtgttgagtctagaggttatattattggtgctccattggctgttgctttgggt
attccttttgttactgctagagttactaagagatttccatcttctttcgttcctgaaggagatg

atttgaagtatttgccaatgtctagatctatcagaaacgattctattccacctagagctagagt

tttgattgttgatgatttcattggtactggttctactatgttggctgctttgagattggctgat

attgttgctgctcaagttgttgaggttttgactgtttgtgatgttgcttctttgggtggtatta

aaatcatcagagaatctgatgatgagatgttcaaggaaactcctatttttactttgatccactt

caaattgtctcctagagaagcagaagagcagttggagttcgtcaacagttacatcacaagaagt

agattgtgagtttgtagccttagaca

>APRT1_opt_Ctag

gaagctagcctcgagccaccatgagtcttgttgaggttttgcctaactattttacattgtctaa

ggattcaccattgagaaagaagttcgagaaagtctataagtggtactctccagctttttctcct

catgatgttccaagattcgctgaagttggtaacattactgaaaatcctgaggttatgagaggta

tcagagatttctttgttgatagatacaagaacttgcaacaaccaatcactcacattttgggttt

tgattctagaggtttcttgttgggtcctatgattgctgttgaattgaacgttccattcgttttg

attagaaaggctaacaagattgctggtgttattattaagtctgagccttacactaaagaatatg

ctgctgagtctgaagagtgtatgactgttagattcggttctttcgataagaactctagagttgt

tttgatcgatgatgttattgctactggtggtactatgttggctggtgttcaattggttgatgct

tgtggtgctactttggttgaggttgctggtattttgggtttgactttcttgaaaggtactcaac

ctgctcatactttcgctggtggtagatactctaacgttccattcgttactttggttgatgaaac

tgttttgtctgatgagaattgtggtgacccattgcaccacaaaggaagtagaattattagttgc

gctgaagccaagaagttgattggtaccagcgaaaacctgt

>APRT2_opt_Ctag

gaagctagcctcgagccaccatgagtcagtatgatgctatccttaccgaaagacacccacacca

cttcacccttgccgacacccacccacttgctaaagaacttcacgctaacatttttggagagtct

gatttgactcatgctaattgtgctcacgtttacgatatttcttctttgactgaaaagccagctt

tgtttagaaaagttattgagttcttgaagtgtagatacgaaactatgggagatactggtcctac

tcatattattggtgttgagtctagaggttatattattggtgctccattggctgttgctttgggt

attccttttgttactgctagagttactaagagatttccatcttctttcgttcctgaaggagatg

atttgaagtatttgccaatgtctagatctatcagaaacgattctattccacctagagctagagt

tttgattgttgatgatttcattggtactggttctactatgttggctgctttgagattggctgat

attgttgctgctcaagttgttgaggttttgactgtttgtgatgttgcttctttgggtggtatta

aaatcatcagagaatctgatgatgagatgttcaaggaaactcctatttttactttgatccactt

caaattgtctcctagagaagcagaagagcagttggagttcgtcaacagttacatcacaagaagt

agattgggtaccagcgaaaacctgta
