# Supplementary material for: Characterization of adenine phosphoribosyltransferase (APRT) activity in Trypanosoma brucei brucei: Only one of the two isoforms is kinetically active
Source: PLoS Negl Trop Dis. 2022 Feb 1;16(2):e0009926. doi: 10.1371/journal.pntd.0009926 (PMC8836349; doi:10.1371/journal.pntd.0009926)
Supplement: S2 Appendix — (PDF) [file pntd.0009926.s002.pdf]

S2 Appendix. Primers used for linearization of plasmids pET-28a(+)::*aprt1-Ntag*, pET-28a(+)::*aprt1-Ctag*, pET-28a(+)::*aprt2-Ntag* and pET-28a(+)::*aprt2-Ctag* prior to cloning into pPICZ vectors.

pPICZ Ntag:

Fwd: 5'-GTACTTCCAAAGCGGTACC-3'

Rev: 5'-TGAGTTTGTAGCCTTAGACATGACTGTTCCTCAG-3'

pPICZ Ctag:

Fwd: 5'-GCTGTCTTGGAACCTAATATG-3'

Rev: 5'-TGTCAGTTTTGGGCCATTTG-3'
