# Supplementary material for: Characterization of adenine phosphoribosyltransferase (APRT) activity in Trypanosoma brucei brucei: Only one of the two isoforms is kinetically active
Source: PLoS Negl Trop Dis. 2022 Feb 1;16(2):e0009926. doi: 10.1371/journal.pntd.0009926 (PMC8836349; doi:10.1371/journal.pntd.0009926)
Supplement: S2 Fig — Vector maps of APRT2-Ntag (left) and APRT2-Ctag (right). P. pastoris expression vector pPICZ maps, showing the aprt2 gene insert position, and location of mCherry tags. (PDF) [file pntd.0009926.s004.pdf]

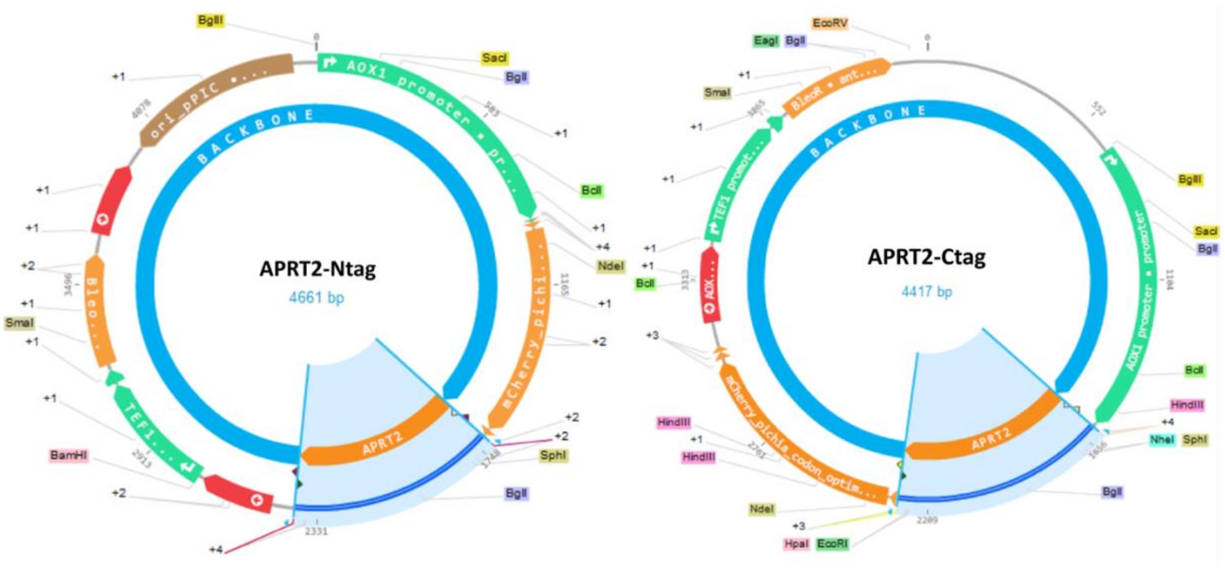

S2 Fig. Vector maps of APRT2-Ntag (left) and APRT2-Ctag (right). *P. pastoris* expression vector pPICZ maps, showing the *aprt2* gene insert position, and location of mCherry tags.
