# Supplementary material for: Characterization of adenine phosphoribosyltransferase (APRT) activity in Trypanosoma brucei brucei: Only one of the two isoforms is kinetically active
Source: PLoS Negl Trop Dis. 2022 Feb 1;16(2):e0009926. doi: 10.1371/journal.pntd.0009926 (PMC8836349; doi:10.1371/journal.pntd.0009926)
Supplement: S3 Fig — The highest expressing clones were selected based on mCherry expression levels (magenta color). (PDF) [file pntd.0009926.s005.pdf]

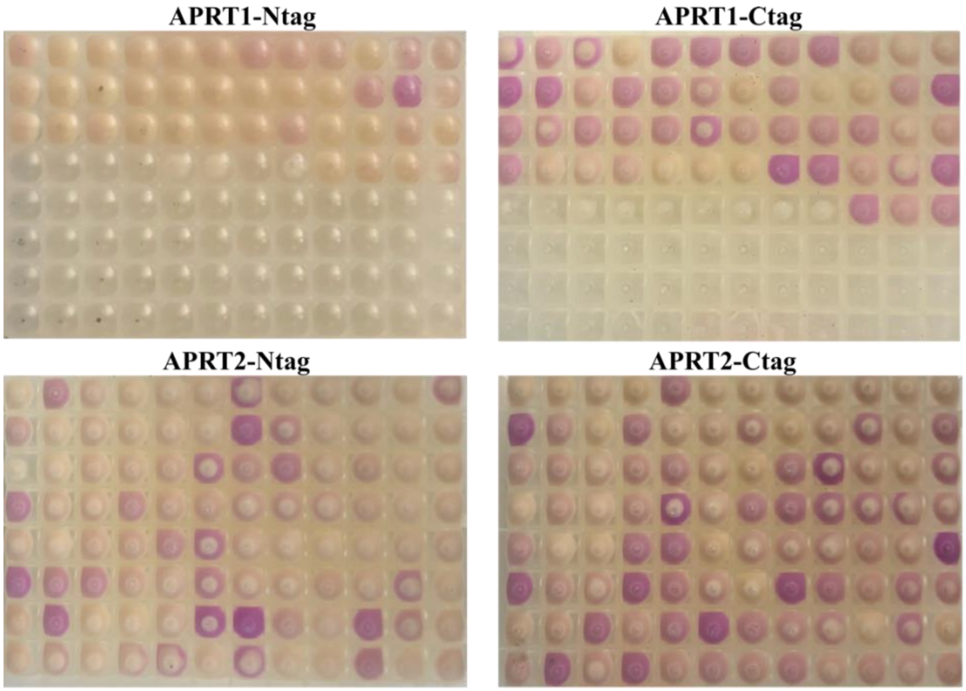

S3 Fig. Small scale expression screen of recombinant APRT1 and APRT2 in *P. pastoris*, co-expressed with mCherry. The highest expressing clones were selected based on mCherry expression levels (magenta color).
