# Supplementary material for: Characterization of adenine phosphoribosyltransferase (APRT) activity in Trypanosoma brucei brucei: Only one of the two isoforms is kinetically active
Source: PLoS Negl Trop Dis. 2022 Feb 1;16(2):e0009926. doi: 10.1371/journal.pntd.0009926 (PMC8836349; doi:10.1371/journal.pntd.0009926)
Supplement: S4 Fig — The scale of the y-axis is significantly smaller for APRT2 when compared to APRT1 (PDF) [file pntd.0009926.s006.pdf]

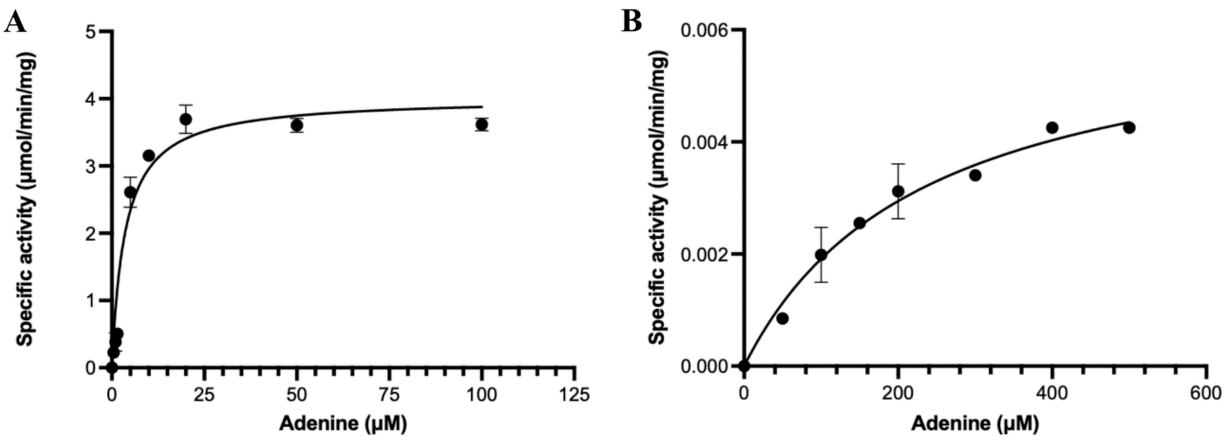

S4 Fig. Initial velocity data of APRT1 and APRT2 at apparent saturating concentrations of PRPP (1mM). The scale of the y-axis is significantly smaller for APRT2 when compared to APRT1.
