# Supplementary material for: Characterization of adenine phosphoribosyltransferase (APRT) activity in Trypanosoma brucei brucei: Only one of the two isoforms is kinetically active
Source: PLoS Negl Trop Dis. 2022 Feb 1;16(2):e0009926. doi: 10.1371/journal.pntd.0009926 (PMC8836349; doi:10.1371/journal.pntd.0009926)
Supplement: S5 Fig — APRT reverse reaction was monitored in presence of adenine deaminase (EC 3.5.4.2) to favor adenine synthesis: AMP+PPi→adenine+PRPP→hypoxanthine+NH3. Forward reaction control shows the elution peak corresponding to adenine (Rt = 4.2 minutes, black line), and reverse reaction control shows the elution peak corresponding to AMP (Rt = 3.6 minutes, blue line). Although the reverse APRT activity is detected, the extent of the conversion of AMP (insert) into adenine is minor, under similar assay conditions, when compared to the forward reaction (Table 1). (PDF) [file pntd.0009926.s007.pdf]

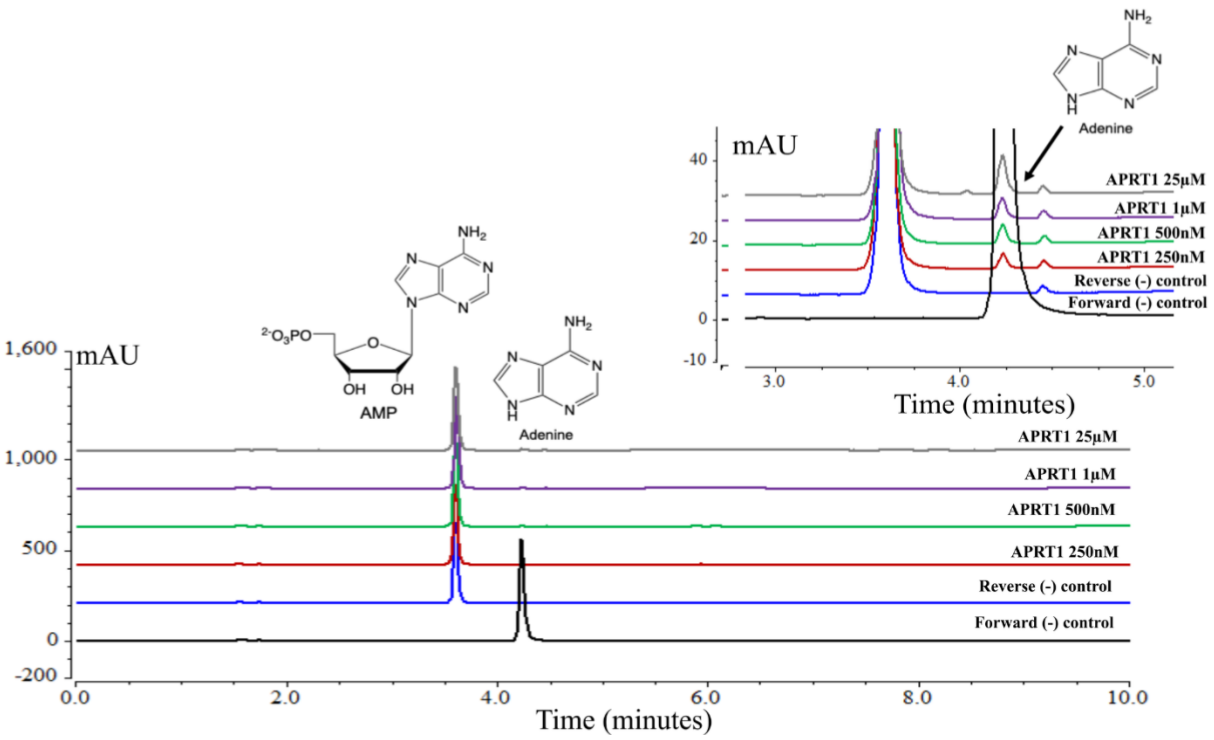

S5 Fig. HPLC chromatograms of APRT1 reverse activity. APRT reverse reaction was monitored in presence of adenine deaminase (EC 3.5.4.2) to favor adenine synthesis: $AMP + PP_i \xrightarrow{2.4.2.7} adenine + PRPP \xrightarrow{3.5.4.2} hypoxanthine + \; NH_3$. Forward reaction control shows the elution peak corresponding to adenine (R_t = 4.2 minutes, black line), and reverse reaction control shows the elution peak corresponding to AMP (R_t = 3.6 minutes, blue line). Although the reverse APRT activity is detected, the extent of the conversion of AMP (insert) into adenine is minor, under similar assay conditions, when compared to the forward reaction (Table 1).
