# Supplementary material for: Characterization of adenine phosphoribosyltransferase (APRT) activity in Trypanosoma brucei brucei: Only one of the two isoforms is kinetically active
Source: PLoS Negl Trop Dis. 2022 Feb 1;16(2):e0009926. doi: 10.1371/journal.pntd.0009926 (PMC8836349; doi:10.1371/journal.pntd.0009926)
Supplement: S6 Fig — (A) Initial velocities of APRT1 at fixed-changed PRPP concentrations, varying substrate adenine. (B) Initial velocities of APRT1 at fixed-changed adenine concentrations, varying substrate PRPP. Data Insets shows double reciprocal plots of A and B. (PDF) [file pntd.0009926.s008.pdf]

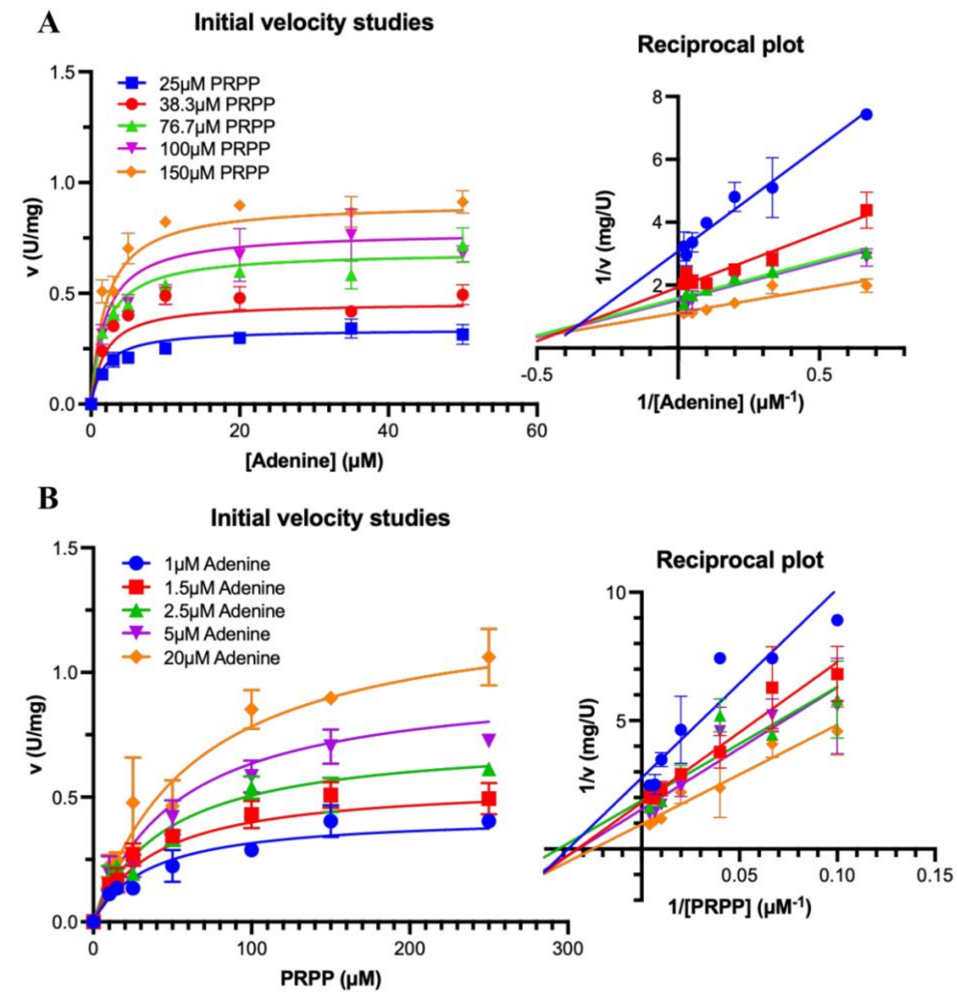

S6 Fig. Bi-substrate kinetics of APRT1 forward reaction. (**A**) Initial velocities of APRT1 at fixed-changed PRPP concentrations, varying substrate adenine. (**B**) Initial velocities of APRT1 at fixed-changed adenine concentrations, varying substrate PRPP. Data Insets shows double reciprocal plots of A and B.
