# Supplementary material for: Characterization of adenine phosphoribosyltransferase (APRT) activity in Trypanosoma brucei brucei: Only one of the two isoforms is kinetically active
Source: PLoS Negl Trop Dis. 2022 Feb 1;16(2):e0009926. doi: 10.1371/journal.pntd.0009926 (PMC8836349; doi:10.1371/journal.pntd.0009926)
Supplement: S7 Fig — An apparent Kia value was obtained with the secondary replot of the initial velocity double reciprocal slopes (right), shown in S6 Fig, where slope = KiaKb/Vmax. Apparent Kia was used as starting parameter for the global fitting of the initial velocity data using Eq (2), as described under Methods. (PDF) [file pntd.0009926.s009.pdf]

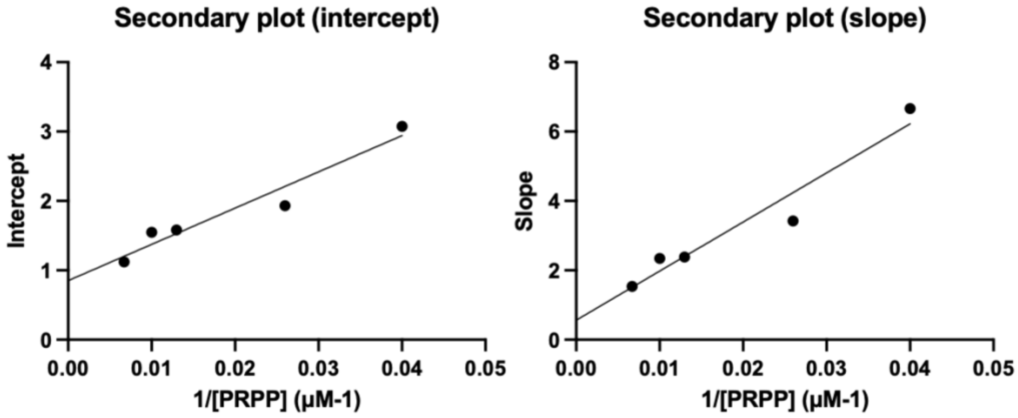

S7 Fig. Secondary reciprocal plots from initial velocity data with fixed and changed PRPP concentrations, varying substrate adenine. An apparent K_{ia} value was obtained with the secondary replot of the initial velocity double reciprocal slopes (right), shown in S5 Fig, where $slope = K_{ia}K_b/V_{\max}$. Apparent K_{ia} was used as starting parameter for the global fitting of the initial velocity data using Eq (2), as described under Methods.
