# Supplementary figures and images for: Characterization of adenine phosphoribosyltransferase (APRT) activity in Trypanosoma brucei brucei: Only one of the two isoforms is kinetically active
Source: PLoS Negl Trop Dis. 2022 Feb 1;16(2):e0009926. doi: 10.1371/journal.pntd.0009926 (PMC8836349; doi:10.1371/journal.pntd.0009926)

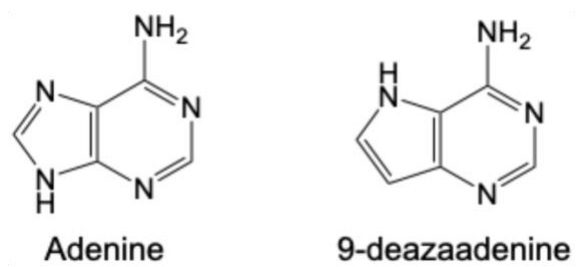

**S8 Fig. Structure of adenine (left) and 9-deazaadenine (right).**

Supplement: S8 Fig — (PDF) [file pntd.0009926.s010.pdf]
