# Supplementary material for: Characterization of adenine phosphoribosyltransferase (APRT) activity in Trypanosoma brucei brucei: Only one of the two isoforms is kinetically active
Source: PLoS Negl Trop Dis. 2022 Feb 1;16(2):e0009926. doi: 10.1371/journal.pntd.0009926 (PMC8836349; doi:10.1371/journal.pntd.0009926)
Supplement: S9 Fig — (PDF) [file pntd.0009926.s011.pdf]

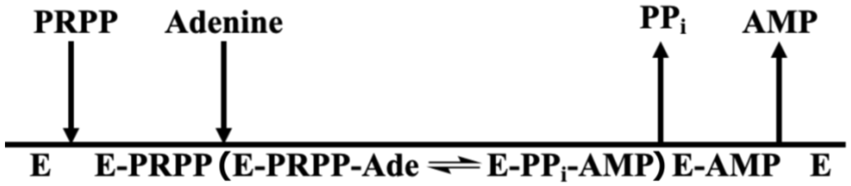

S9 Fig. Ordered Bi Bi kinetic mechanism of APRT1 as obtained from initial velocity and dead-end inhibition studies.
