# Supplementary material for: Characterization of adenine phosphoribosyltransferase (APRT) activity in Trypanosoma brucei brucei: Only one of the two isoforms is kinetically active
Source: PLoS Negl Trop Dis. 2022 Feb 1;16(2):e0009926. doi: 10.1371/journal.pntd.0009926 (PMC8836349; doi:10.1371/journal.pntd.0009926)
Supplement: S10 Fig — The elution volume of APRT1 (blue) and APRT2 (red) suggests both APRTs form homodimers and the SDS-PAGE analysis of eluted fractions illustrates the purity of the samples. The right upper figure shows the calibration curve (blue) for determining the oligomeric states of APRT1 (red) and APRT2 (green). (PDF) [file pntd.0009926.s012.pdf]

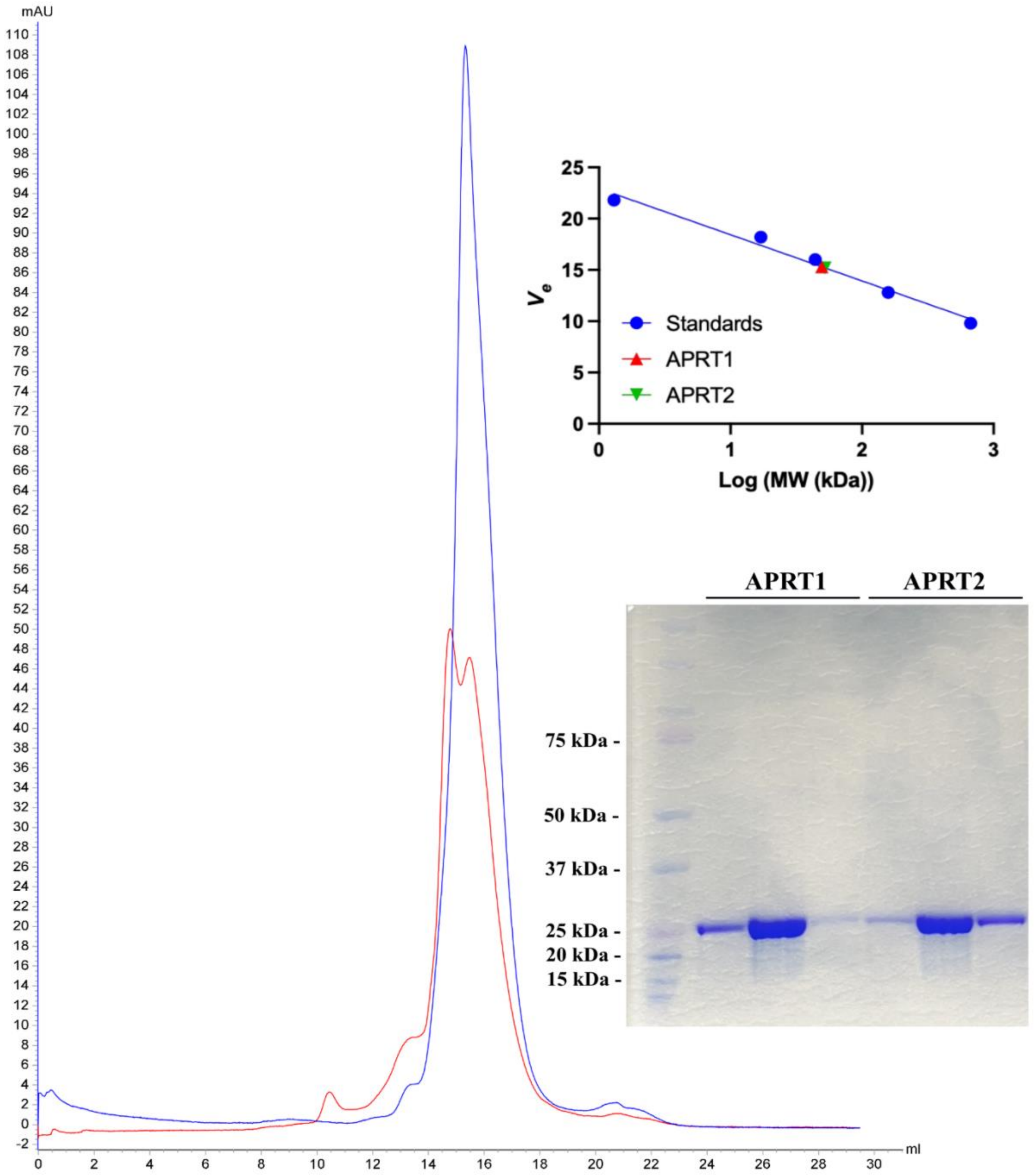

S10 Fig. Size exclusion chromatography of APRT1 and APRT2. The elution volume of APRT1 (blue) and APRT2 (red) suggests both APRTs form homodimers and the SDS-PAGE analysis of eluted fractions illustrates the purity of the samples. The right upper figure shows the calibration curve (blue) for determining the oligomeric states of APRT1 (red) and APRT2 (green).
