# Supplementary material for: Characterization of adenine phosphoribosyltransferase (APRT) activity in Trypanosoma brucei brucei: Only one of the two isoforms is kinetically active
Source: PLoS Negl Trop Dis. 2022 Feb 1;16(2):e0009926. doi: 10.1371/journal.pntd.0009926 (PMC8836349; doi:10.1371/journal.pntd.0009926)
Supplement: S11 Fig — The amino acid residues conserved among all APRTs are highlighted in blue–reflecting the lower sequence conservation on the Type I phosphoribosyltransferases family of enzymes. The β-phosphoryl binding sequence is underlined in black; the catalytic loop sequence is underlined in green, and the PRPP binding domain is underlined in red. The C-terminal extension characteristic of the long APRTs is underlined in gray. The C-terminal glycosomal signaling peptide on TbbAPRT2 is boxed in orange. The conserved residue arginine 82 (TbbAPRT1 numbering–black dot) participates on binding of PRPP’s β-phosphoryl group. Arginine 102 (gray dot) composes the active site of the adjacent subunit–highlighting the importance of the dimeric quaternary structure for catalytic activity. Tyrosine 121 (red dot) is located on the tip of the loop that closes over the active site after both substrates are bound, being essential for APRTs to adopt their catalytic conformation. For more details on the amino acid residues identified as essential for APRTs substrates binding and catalysis, refer [65,93,94]. (PDF) [file pntd.0009926.s013.pdf]

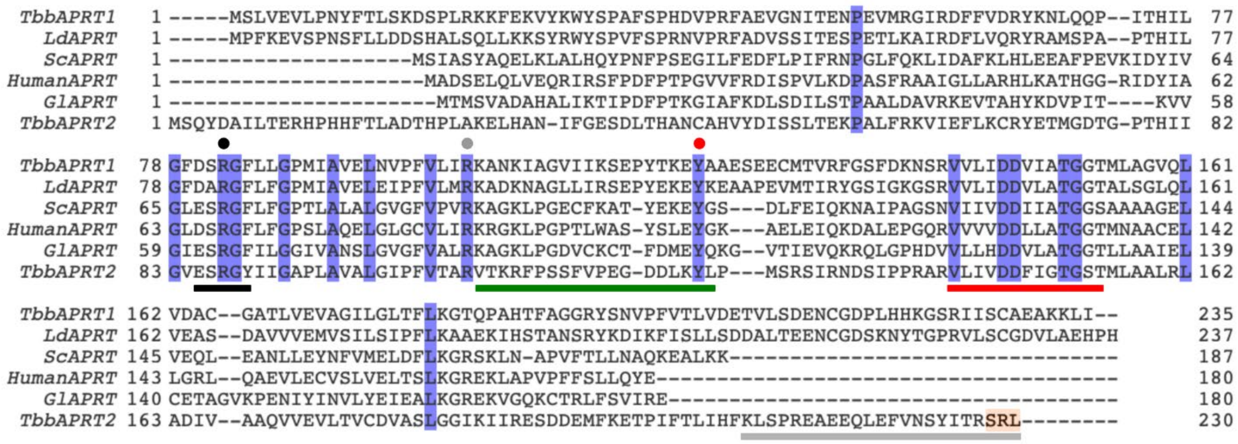

S11 Fig. Sequence alignment of APRT1, APRT2, and previously characterized APRTs. The amino acid residues conserved among all APRTs are highlighted in blue – reflecting the lower sequence conservation on the type I phosphoribosyltransferases family of enzymes. The β-phosphoryl binding sequence is underlined in black; the catalytic loop sequence is underlined in green, and the PRPP binding domain is underlined in red. The C-terminal extension characteristic of the long APRTs is underlined in gray. The C-terminal glycosomal signaling peptide on TbbAPRT2 is boxed in orange. The conserved residue arginine 82 (TbbAPRT1 numbering – black dot) participates on binding of PRPP's β-phosphoryl group. Arginine 102 (gray dot) composes the active site of the adjacent subunit – highlighting the importance of the dimeric quaternary structure for catalytic activity. Tyrosine 121 (red dot) is located on the tip of the loop that closes over the active site after both substrates are bound, being essential for APRTs to adopt their catalytic conformation. For more details on the amino acid residues identified as essential for APRTs substrates binding and catalysis, refer to [66, 94-95].
