# Supplementary material for: Characterization of adenine phosphoribosyltransferase (APRT) activity in Trypanosoma brucei brucei: Only one of the two isoforms is kinetically active
Source: PLoS Negl Trop Dis. 2022 Feb 1;16(2):e0009926. doi: 10.1371/journal.pntd.0009926 (PMC8836349; doi:10.1371/journal.pntd.0009926)
Supplement: S12 Fig — APRT2 was tested for activity as a potential HGXPRT, EC 2.4.2.8 (A, B, C); OPRT, EC 2.4.2.10 (D), UPRT, EC 2.4.2.9 (E), and PRPP synthetase, EC 2.7.6.1 (F). No enzymatic activity could be detected under the assay conditions described in the Methods. (PDF) [file pntd.0009926.s014.pdf]

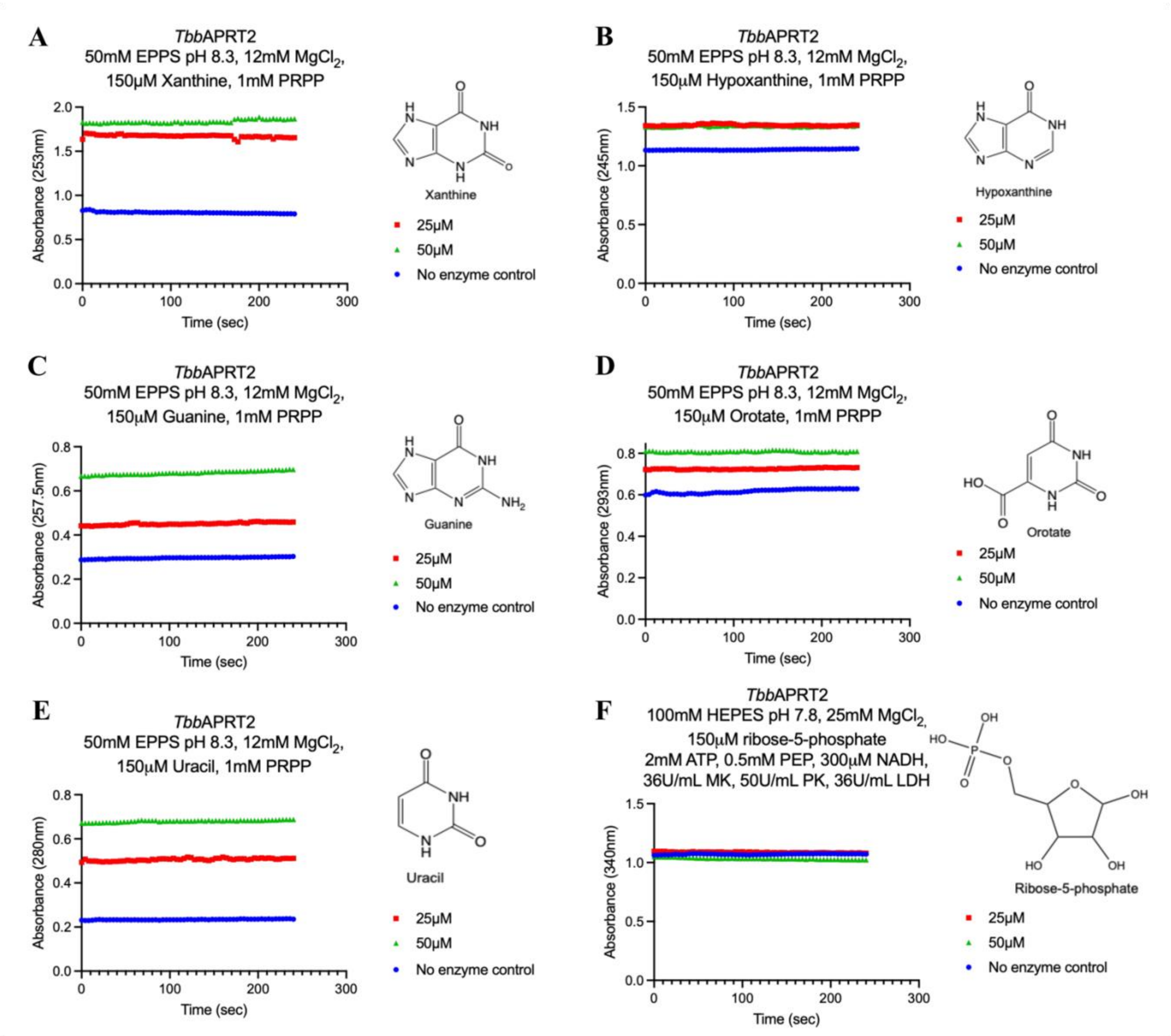

S12 Fig. APRT2 Type 1 PRTase activity tests using alternative N-containing nucleophiles – part 1. APRT2 was tested for activity as a potential HGXPRT, EC 2.4.2.8 (**A**, **B**, **C**); OPRT, EC 2.4.2.10 (**D**), UPRT, EC 2.4.2.9 (**E**), and PRPP synthetase, EC 2.7.6.1 (**F**). No enzymatic activity could be detected under the assay conditions described in the Methods.
