# Supplementary material for: Characterization of adenine phosphoribosyltransferase (APRT) activity in Trypanosoma brucei brucei: Only one of the two isoforms is kinetically active
Source: PLoS Negl Trop Dis. 2022 Feb 1;16(2):e0009926. doi: 10.1371/journal.pntd.0009926 (PMC8836349; doi:10.1371/journal.pntd.0009926)
Supplement: S13 Fig — APRT2 was tested for activity as a potential adenosine phosphorylase, EC 2.4.2.1 (A), and 5’-nucleotidase, EC 3.1.3.5 (B). No enzymatic activity could be detected under the assay conditions described in the Methods. (PDF) [file pntd.0009926.s015.pdf]

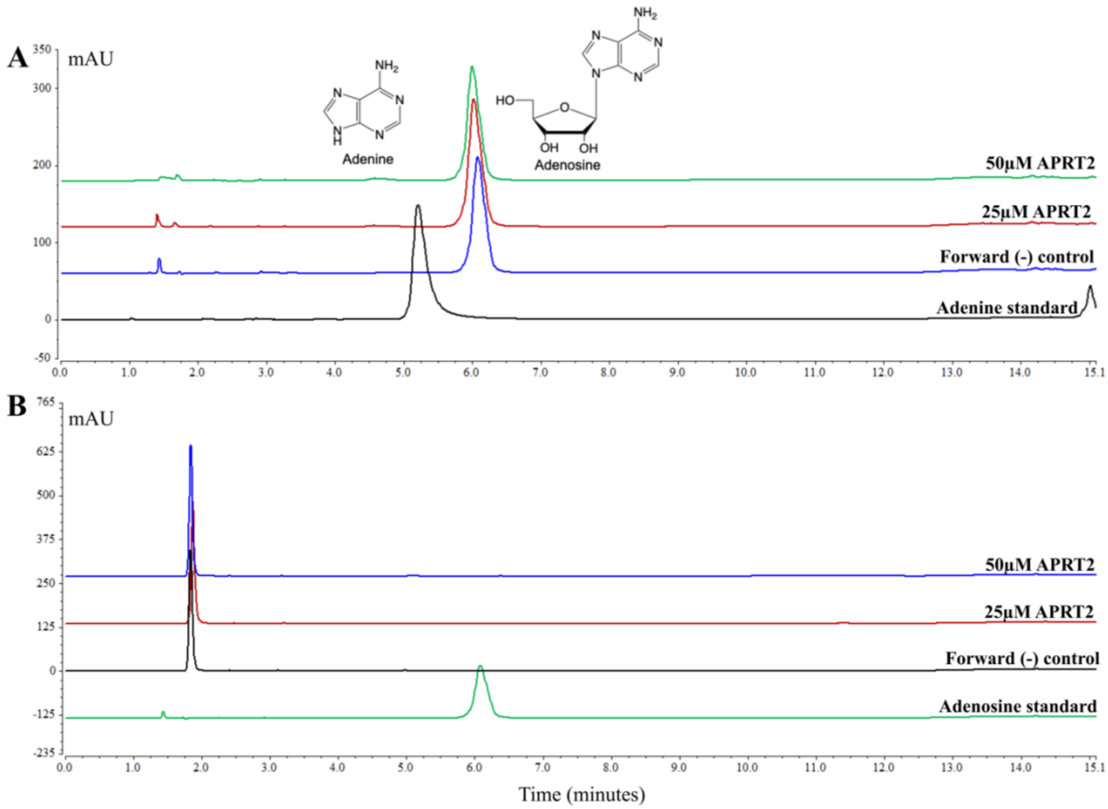

S13 Fig. APRT2 Type 1 PRTase activity tests using alternative N-containing nucleophiles – part 2. APRT2 was tested for activity as a potential adenosine phosphorylase, EC 2.4.2.1 (**A**), and 5'-nucleotidase, EC 3.1.3.5 (**B**). No enzymatic activity could be detected under the assay conditions described in the Methods.
