# Supplementary material for: Characterization of adenine phosphoribosyltransferase (APRT) activity in Trypanosoma brucei brucei: Only one of the two isoforms is kinetically active
Source: PLoS Negl Trop Dis. 2022 Feb 1;16(2):e0009926. doi: 10.1371/journal.pntd.0009926 (PMC8836349; doi:10.1371/journal.pntd.0009926)
Supplement: S14 Fig — Cyanogen bromide selectively cleaves peptides at the C-terminus of reduced methionine residues. APRT2 contains five Met residues and when all Met residues are reduced, 6 fragments are produced. The molecular weight of all 6 fragments expected for APRT2-Ntag (A) and APRT2-Ctag (B) are depicted on each fragment. (PDF) [file pntd.0009926.s016.pdf]

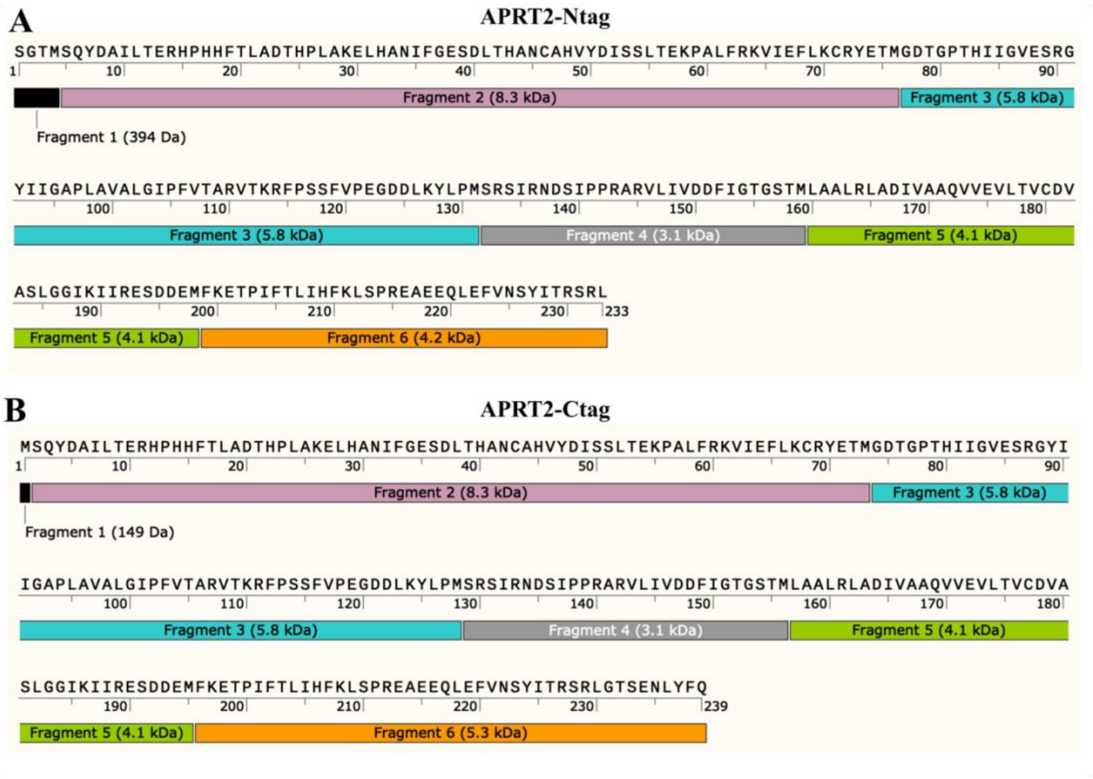

S14 Fig. Expected fragments of APRT2 with no MetO modifications treated with cyanogen bromide. Cyanogen bromide selectively cleaves peptides at the C-terminus of reduced methionine residues. APRT2 contains five Met residues and when all Met residues are reduced, 6 fragments are produced. The molecular weight of all 6 fragments expected for APRT2-Ntag **(A)** and APRT2-Ctag **(B)** are depicted on each fragment.
