# Supplementary material for: Characterization of adenine phosphoribosyltransferase (APRT) activity in Trypanosoma brucei brucei: Only one of the two isoforms is kinetically active
Source: PLoS Negl Trop Dis. 2022 Feb 1;16(2):e0009926. doi: 10.1371/journal.pntd.0009926 (PMC8836349; doi:10.1371/journal.pntd.0009926)
Supplement: S15 Fig — The crystal structure of APRT1 shows 2 methionine residues located near the PRPP binding site (M129 and M155, upper right side) and one methionine residue located near the subunit interface (M85, lower right side). APRT1 subunits are colored in green and blue ribbons. Ligands (adenine and ribose-5-phosphate), as well as the methionine sidechains, are represented as sticks. (PDF) [file pntd.0009926.s017.pdf]

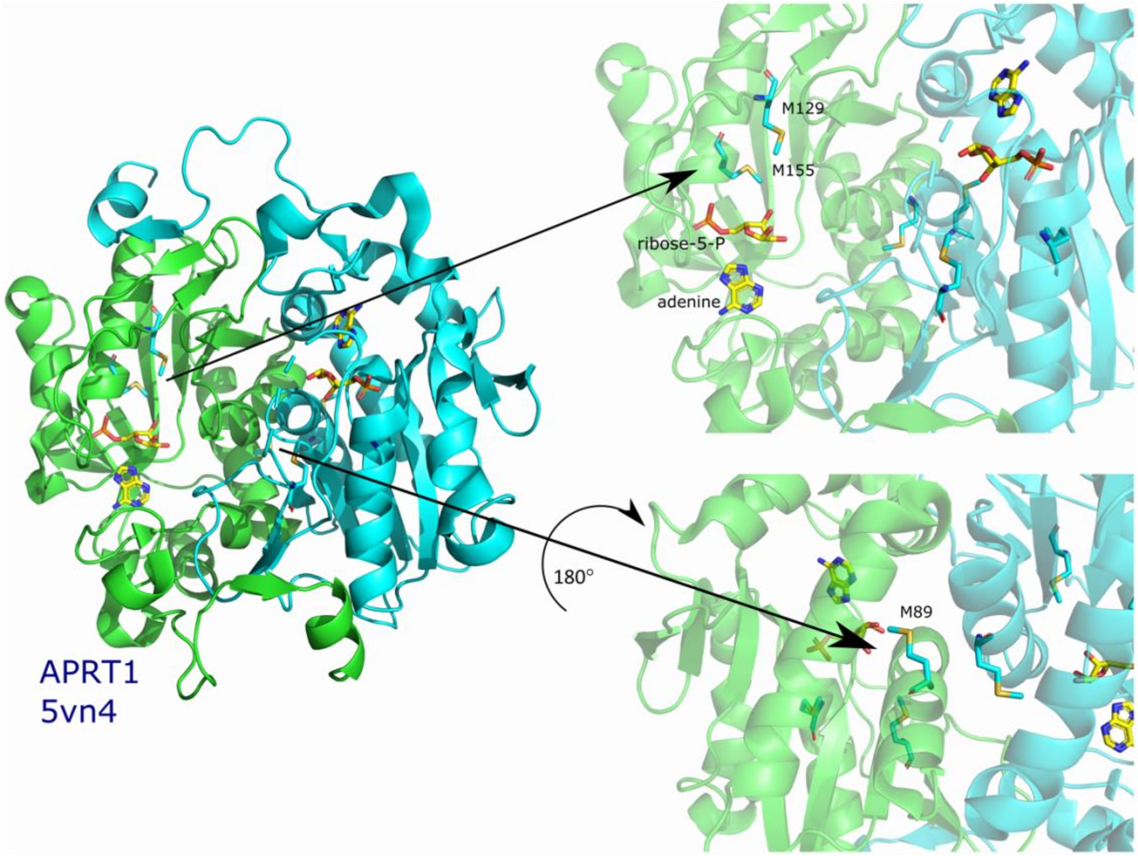

S15 Fig. Location of methionine residues on APRT1 (PDB code 5VN4). The crystal structure of APRT1 shows 2 methionine residues located near the PRPP binding site (M129 and M155, upper right side) and one methionine residue located near the subunit interface (M85, lower right side). APRT1 subunits are colored in green and blue ribbons. Ligands (adenine and ribose-5-phosphate), as well as the methionine sidechains, are represented as sticks.
