# Supplementary material for: Characterization of adenine phosphoribosyltransferase (APRT) activity in Trypanosoma brucei brucei: Only one of the two isoforms is kinetically active
Source: PLoS Negl Trop Dis. 2022 Feb 1;16(2):e0009926. doi: 10.1371/journal.pntd.0009926 (PMC8836349; doi:10.1371/journal.pntd.0009926)
Supplement: S16 Fig — APRT reverse reaction was monitored in presence of adenine deaminase (EC 3.5.4.2) to favor adenine synthesis: AMP+PPi→adenine+PRPP→hypoxanthine+NH3. None of the three APRT2 M to Q mutants shows APRT reverse activity, either in 30 min (top graph) or overnight (bottom graph) incubations, as indicated by the invariable AMP peak and absence of formation of adenine. Forward reaction controls show the elution peak corresponding to adenine (black lines), and reverse reaction controls show the elution peak corresponding to AMP (blue lines). The eluted peak at 6.2 minutes was present in all APRT2 assay fractions (Fig 7) and could not be satisfactorily isolated and identified under current assay conditions. (PDF) [file pntd.0009926.s018.pdf]

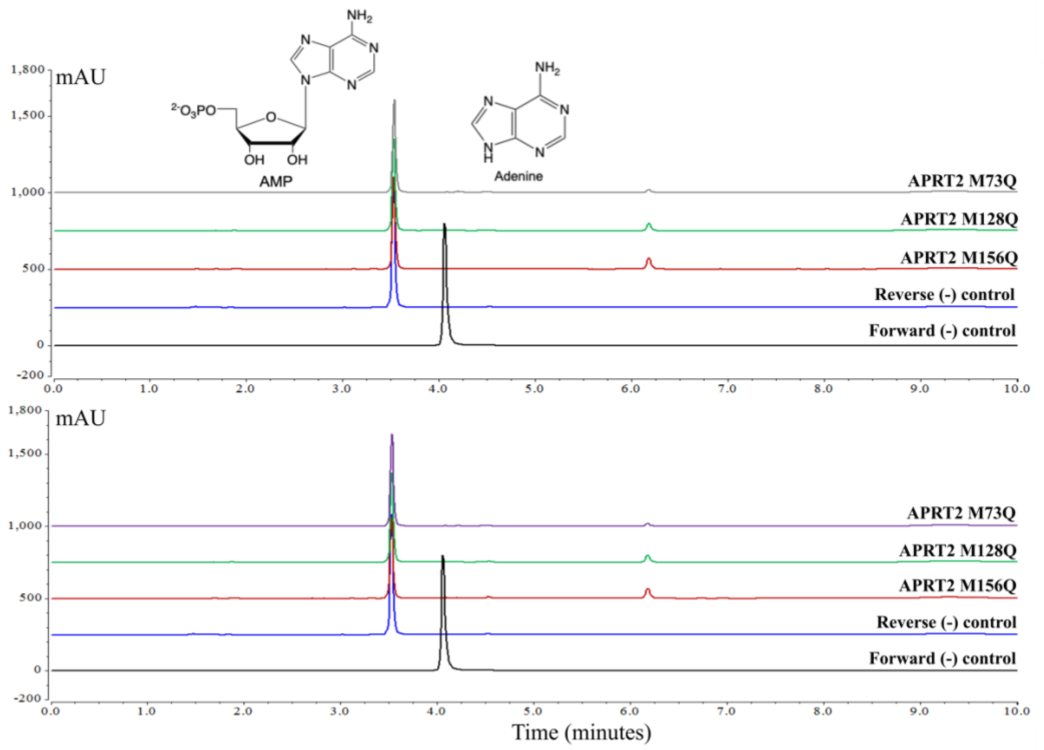

S16 Fig. HPLC chromatograms of APRT2 M to Q mutants' activity. APRT reverse reaction was monitored in presence of adenine deaminase (EC 3.5.4.2) to favor adenine synthesis: $AMP + PP_i \xrightarrow{2.4.2.7} adenine + PRPP \xrightarrow{3.5.4.2} hypoxanthine + NH_3$. None of the three APRT2 M to Q mutants shows APRT reverse activity, either in 30 min (top graph) or overnight (bottom graph) incubations, as indicated by the invariable AMP peak and absence of formation of adenine. Forward reaction controls show the elution peak corresponding to adenine (black lines), and reverse reaction controls show the elution peak corresponding to AMP (blue lines). The eluted peak at 6.2 minutes was present in all APRT2 assay fractions (Fig 7) and could not be satisfactorily isolated and identified under current assay conditions.
